# tRNA Metabolism and Lung Cancer: Beyond Translation

**DOI:** 10.3389/fmolb.2021.659388

**Published:** 2021-09-30

**Authors:** Meng Bian, Shiqiong Huang, Dongsheng Yu, Zheng Zhou

**Affiliations:** ^1^ Department of Chinese Medicine, The First Affiliated Hospital of Zhengzhou University, Zhengzhou, China; ^2^ Department of Pharmacy, The First Hospital of Changsha, Changsha, China

**Keywords:** lung cancer, pathogenesis, therapeutics, tRNA derivatives, tRNA modifications, tRNA aminoacylation

## Abstract

Lung cancer, one of the most malignant tumors, has extremely high morbidity and mortality, posing a serious threat to global health. It is an urgent need to fully understand the pathogenesis of lung cancer and provide new ideas for its treatment. Interestingly, accumulating evidence has identified that transfer RNAs (tRNAs) and tRNA metabolism–associated enzymes not only participate in the protein translation but also play an important role in the occurrence and development of lung cancer. In this review, we summarize the different aspects of tRNA metabolism in lung cancer, such as tRNA transcription and mutation, tRNA molecules and derivatives, tRNA-modifying enzymes, and aminoacyl-tRNA synthetases (ARSs), aiming at a better understanding of the pathogenesis of lung cancer and providing new therapeutic strategies for it.

## Introduction

Lung cancer is one of the most malignant tumors and a cause for cancer-related deaths globally, accounting for about 18% of all cancer deaths (Sung et al., 2021). The majority of lung cancers are non–small cell lung cancers (NSCLCs), among which the most common subtypes are adenocarcinoma and squamous cell carcinoma (Herbst et al., 2018). The early symptoms of lung cancer are usually mild or even without any discomfort. Most of the patients with lung cancer are diagnosed in the advanced stage, which is generally associated with daunting metastases (Popper, 2016; Yousefi et al., 2017). Furthermore, research data found that the 5-year survival rate of patients with lung cancer distant metastasis was only 18.6% (Fitzmaurice et al., 2017). This poor prognosis emphasizes the importance of diagnosis and treatment before overt metastases develop. At the same time, due to the heterogeneity of lung cancer, understanding its pathogenesis is crucial for developing effective treatments (Nicoś et al., 2020; Hou et al., 2021).

**FIGURE 1:**
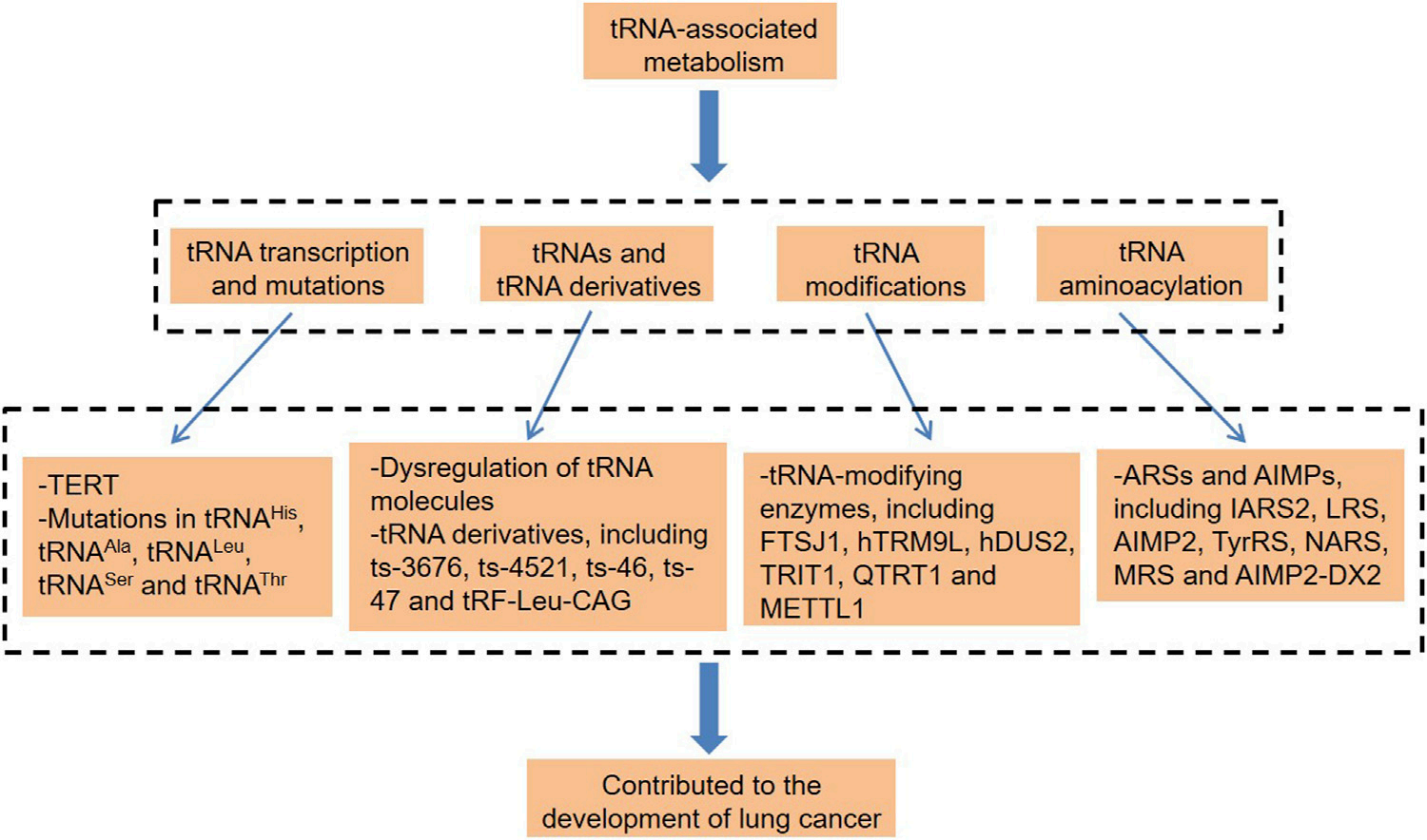
tRNA-associated metabolism and lung cancer. tRNA, transfer RNA; TERT, telomerase reverse transcriptase; hTRM9L, tRNA methyltransferase 9-like; TRIT1, tRNA isopentenyltransferase 1; QTRT1, tRNA-ribosyltransferase 1; METTL1, methyltransferase-like 1; ARSs, aminoacyl-tRNA synthetases; AIMPs, ARS-interacting multifunctional proteins; IARS2, isoleucyl-tRNA synthetase 2; LRS, leucyl-tRNA synthetase; TyrRS, tyrosine-tRNA synthetase; NARS, asparaginyl-tRNA synthetase; MRS, methionyl-tRNA synthetase; and AIMP2-DX2, AIMP2 lacking exon 2.

Not participating in protein-coding and small noncoding RNAs (sncRNAs) is a phenomenon that exists widely and plays a widespread and important role in organisms (Zhao et al., 2018; Li et al., 2018b; Zhou et al., 2019; Ying et al., 2021). Transfer RNAs (tRNAs) are among the most abundant sncRNAs, which are widely found in organisms and account for about 4–10% of all cellular RNAs (Kirchner and Ignatova, 2015). Traditionally, tRNAs are involved in protein translation and have a basic function of carrying and transporting amino acids, which is key to the high efficiency and accuracy of protein synthesis (Grewal, 2015; Huang et al., 2018). In recent years, there has been growing evidence that tRNAs contribute to the pathogenic process of various cancers (Ignatova et al., 2020; Zhang et al., 2020b; Pan et al., 2021; Yang et al., 2021). tRNAs can be specifically spliced into regulatory fragments under the effect of hypoxia and other stress conditions, which promotes lung cancer cell proliferation and cell cycle (Shao et al., 2017). In some cases, the mutations of mitochondria-encoded tRNA (mt-tRNA) were pathogenic and highly likely to promote the carcinogenesis of lung cancer (Wang et al., 2015; He et al., 2016). Besides, tRNA modification is also inseparable from lung cancer (He et al., 2020). Thus, this article focuses on the different types of tRNA-associated dysregulation in lung cancer (Figure 1), which provide new ideas for the diagnosis and treatment of this disease.

## Roles of tRNA Metabolism in Lung Cancer

### tRNA Transcription and Mutations in Lung Cancer

RNA polymerase III (Pol III) is mainly responsible for the transcription of tRNA and 5S rRNA in eukaryotes (Wang and Gerber, 2020). Of note, the transcription of tRNAs by RNA Pol III was influenced by all kinds of oncogenes and tumor-suppressor genes (Rollins et al., 2007; Johnson et al., 2008). The transcription of Pol III in healthy cells is inhibited by tumor suppressors (White, 2008). However, this limitation is compromised during cell transformation, and oncogene products exacerbated this problem, which stimulated the output of Pol III (White, 2008). Interestingly, there is a significant positive correlation between telomerase reverse transcriptase (TERT) and tRNA levels in cancer. TERT directly binds to the Pol III subunit RPC32 and upregulates the recruitment of chromatin, resulting in an increase in the occupancy rate of Pol III on the tRNA genes (Khattar et al., 2016). TERT is significantly enriched at tRNA^Met^, tRNA^Arg^, and tRNA^Lys^ genes, regulating the expression of these tRNAs, thus controlling the rate of protein synthesis in cancer cells and promoting tumorigenesis to a certain extent (Khattar et al., 2016).

Mutations of tRNA usually occur in mitochondria due to the lack of protective histones, introns, and effective DNA repair systems in mitochondrial DNA (mtDNA) (Wallace, 2015). These mutations in certain mt-tRNAs, such as tRNA^His^, tRNA^Ala^, tRNA^Leu^, tRNA^Ser^, and tRNA^Thr^, have strong pathogenicity and are highly likely to be related to the carcinogenesis of lung cancer (Wang et al., 2015; He et al., 2016). mtDNA mutation destroys the secondary structure of tRNA itself and affects the tRNA posttranscriptional modifications and aminoacylation, which causes a decrease in the mitochondrial protein synthesis and the inability to meet the respiratory phenotype and the threshold of ATP required by normal cells, thereby promoting tumorigenesis (Lu et al., 2009).

Of note, recent studies found that the newly designed β32_33 peptide could penetrate the mitochondrial membrane and improve the viability of the cells containing mt-tRNA^Leu(UUR)^ m.3243A>G and mt-tRNA^Lys^ m.8344A>G mutations by stabilizing the structure of mt-tRNA mutants (Perli et al., 2020). Moreover, mitochondrially targeted zinc finger nucleases (mtZFNs) shifted the heteroplasmy of the mt-tRNA^ALA^ m.5024C>T mutant, thereby rescuing mitochondrial functions (Gammage and Viscomi, 2018). These findings indicate that correcting the pathogenic mt-tRNA mutations can rescue disease phenotypes, which provides new ideas for the treatment of lung cancer. In all, both tRNA transcription and mutation are involved in the pathogenesis of lung cancer.

### tRNA Molecules and Derivatives in Lung Cancer

In addition, the dysregulation of tRNA levels is closely related to the prognosis of lung cancer. Analyzing the expression level of tRNA in lung adenocarcinoma tissues and paracarcinoma tissues by the tRNA RT-qPCR array, Kuang *et al*. found that there were differences in many tRNA levels, such as tRNA^Asn^, tRNA^Ile^, and tRNA^Leu^ (Kuang et al., 2019). Through further analysis of the correlation between the tRNA expression and clinicopathological characteristics, they unraveled that the expression of three tRNAs, tRNA^Ile^, tRNA^Pro^, and tRNA^Lys^ was related to tumor differentiation, and patients with a higher expression of mt-tRNA^Glu^ and tRNA^Tyr^ and lower expression of tRNA^Thr^ and tRNA^Asn^ had a higher risk of relapse (Kuang et al., 2019). Moreover, the levels of tRNA^Lys^, mt-tRNA^Ser^, and tRNA^Tyr^ were associated with cancer-specific survival (Kuang et al., 2019). These tRNAs were used as variables to construct a prognostic model of lung adenocarcinoma and could accurately predict cancer-specific survival in patients with lung adenocarcinoma (Kuang et al., 2019).

The initial transcription product of RNA Pol III is a precursor of tRNA (pre-tRNA), which must undergo a series of complex biological processes before being converted into mature tRNA (Vannini and Cramer, 2012). Under the effect of sex hormones, hypoxia, and other stress conditions, pre-tRNAs and mature tRNAs are found to be significant signaling molecules, which are broken down into tRNA derivatives (Thompson et al., 2008; Veneziano et al., 2016). Under stress, tRNA breakdown products are more frequently found in tumor tissues (Dong et al., 2020; Farina et al., 2020; Wang et al., 2021b). According to the different cleavage sites, tRNA derivatives are mainly divided into two types: tRNA-derived stress-induced RNAs (tiRNAs) generated by cleaving the anticodon loops of the mature tRNAs (Saikia and Hatzoglou, 2015; Shigematsu and Kirino, 2017) and tRNA-derived fragments (tRFs) formed by cutting the mature or precursor tRNAs in the D-loop, T-loop, and other positions (Keam and Hutvagner, 2015; Pekarsky et al., 2016). Strikingly, increasing evidence argued that tRNA derivatives were dysregulated in lung cancer (Pekarsky et al., 2016; Balatti et al., 2017). ts-3676 and ts-4521, which were derived from tRNA^Thr^ and tRNA^Ser^, respectively, could not only interact with the Argonaute proteins Ago1 and Ago2 to act as microRNAs but also interact with the P-element–induced wimpy testis (Piwi)–like protein 2 (PiwiL2) to serve as Piwi-interacting small RNAs (piRNAs) (Pekarsky et al., 2016). It was worth noting that these two tsRNAs were significantly downregulated and mutated in lung cancer tissues than normal lung tissues (Pekarsky et al., 2016). In addition, using pathway analysis software to evaluate the roles of ts-4521 in cancer, Balatti et al. found that the downregulation of ts-4521 was related to the cell proliferation and apoptosis-related signal pathways (Balatti et al., 2017). Moreover, the overexpression of ts-46 and ts-47 had a strong inhibitory effect on cell colony formation in lung cancer cells (Balatti et al., 2017).

Another study found that tRFs could effectively regulate kinase activity. tRF-Leu-CAG, derived from tRNA^Leu(CAG)^, regulated the aurora kinase A (AURKA) activity in NSCLC, thereby mediating cell proliferation and cell cycle progression (Shao et al., 2017). AURKA, a serine–threonine kinase, was related to the maturation and separation of the centrosome and regulated the assembly and stability of the spindle, thus playing an important role in mitosis. The overexpression of tRF-Leu-CAG enormously increased the activity of AURKA and promoted the cell proliferation and G0/G1 cell cycle progression in NSCLC, which would be conducive to the deterioration of cancer (Shao et al., 2017). In contrast, AURKA was significantly downregulated in H1299 cells transfected with the tRF-Leu-CAG inhibitor (tRFi), indicating that this tRF might be a potential target for the treatment of lung cancer (Shao et al., 2017). In addition, Chiou *et al*. have demonstrated that antisense oligonucleotides can induce the silencing of tRNA fragments (Chiou et al., 2018). Therefore, it can be speculated that blocking tRNA fragments with oncogenic activity through ASO may be a potential strategy for the treatment of lung cancer.

Various RNA species are released into the extracellular space in the form of extracellular vesicles (EVs) or complexes with proteins, collectively known as extracellular RNA (exRNA) (Happel et al., 2020). Interestingly, exRNAs could not only serve as potential biomarkers for lung cancer (Wang et al., 2020) but also could act as signaling molecules to regulate tumorigenesis (Li et al., 2019). Among them, tRNAs and tRFs are considered to be one of the most abundant RNA components in the extracellular compartment (Torres and Martí, 2021). It is worth noting that the abundance of tRFs in plasma EVs from lung squamous cell carcinoma patients was higher than that from lung adenocarcinoma and healthy individuals, indicating that ex-tRFs might contribute to the development of lung squamous cell carcinoma (Li et al., 2018a). Taken together, the dysregulation of tRNAs and tRNA derivatives has a stake in the pathogenesis of lung cancer.

### tRNA-Modifying Enzymes in Lung Cancer

There are always various modifications after the transcription of tRNAs, which affect not only the stability of tRNAs and codon recognition but also the stability of tRNA transcripts (Martinez et al., 2017; Guo and Ng, 2019; Kimura et al., 2020; Tavares et al., 2021). The number and type of individual tRNA modifications are different, and mammalian cytoplasmic tRNA is estimated to carry 13-14 modifications on an average (Chan et al., 2010). The tRNA modification patterns produced under different environmental stresses are different, indicating that tRNA modification plays a regulatory role in the cellular response to stress (Chan et al., 2010). The pathogenesis of cancer changes the tRNA modification chemistry, which occurs after oxidative stress (Endres et al., 2019). Increasing evidence argues that tRNA modification and corresponding tRNA-modifying enzymes not only play an important role in translation but also are important signal molecules in the pathogenesis of cancer (Rashad et al., 2020; Rosselló-Tortella et al., 2020; Zhang et al., 2020c). Dong et al. found that the modification profiles of tRNA in rapidly proliferating cancer cells were roughly the same, indicating that there was a proliferation-related modification regulation in rapidly proliferating cancer cells, and tRNA had a positive regulatory effect in rapidly proliferating cancer cells (Dong et al., 2016). However, the roles of these tRNA modifications in rapidly proliferating cells remain to be further studied.

Notably, in recent studies, it has been found that tRNA modification was regulated by FTSJ1 in lung cancer, which had a tumor-suppressor effect (He et al., 2020). Eighteen types of tRNA modifications and up to 7 tRNA modification genes in NSCLC tumor tissues were significantly downregulated compared with normal tissues, of which the expression level of 2′-O-methyladenosine (Am) modification was the lowest (He et al., 2020). Further research showed that the amount of Am in tRNAs was significantly related to the expression of FTSJ1, which exerted significant tumor suppressor ability via interacting with DNA damage-regulated autophagy modulator 1 (DRAM1) (He et al., 2020). Similarly, another study indicated that tRNA methyltransferase 9-like (hTRM9L) attenuated the cell cycle by downregulating cyclin D1 and restricted the migration and invasion potential by changing the expression of cadherin in lung cancer (Wang et al., 2018). However, hTRM9L was significantly downregulated in lung cancer tissues, which was closely related to the poor prognosis of patients and was an independent prognostic factor for lung cancer patients (Wang et al., 2018). Collectively, the tRNA-modifying enzymes FTSJ1 and hTRM9L act as tumor suppressors, and measures that can promote their overexpression may be effective in treating lung cancer.

Additionally, some tRNA-modifying enzymes promoted the development of lung cancer (Kato et al., 2005; Coll-SanMartin and Davalos, 2021). hDUS2, a homolog of yeast and bacterial tRNA-dihydrouridine synthases (DUSs), was highly expressed in NSCLC samples, and its high levels were related to the poor prognosis of lung cancer patients (Kato et al., 2005). This enzyme facilitated the formation of dihydrouridine in tRNAs and increased the translation efficiency by interacting with glutamyl-prolyl-tRNA synthetase (EPRS), thereby contributing to tumorigenesis. Significantly, NSCLC cells transfected with si-*hDUS2*-#2 showed a decrease in dihydrouridine levels and growth inhibition, suggesting that the selective inhibition of hDUS2 might have the potential to treat NSCLC (Kato et al., 2005). Coll-SanMartin et al. observed that tRNA isopentenyltransferase 1 (TRIT1) catalyzed the N^6^-isopentenyladenosine (i^6^A) modification at the 37th position of tRNAs, and it showed gene amplification–related overexpression in small cell lung cancer (Coll-SanMartin and Davalos, 2021). Importantly, cancer cells with TRIT1 gene amplification were more sensitive to the drug arsenic trioxide, which provided a theoretical basis for the clinical treatment of such small cell lung cancer patients.

### ARSs in Lung Cancer

It is recognized that tRNA can combine with its homologous amino acids through ARS-mediated aminoacylation, thus transporting amino acids to the ribosomes to participate in protein synthesis (Kwon et al., 2019; Zhou et al., 2020b). In mammalian cells, ARSs can not only exist in their free form in the cytoplasm but also interact with three ARS-interacting multifunctional proteins (AIMPs) to form a multiple tRNA synthetase complex (MSC) (Zhou et al., 2020a). Previously, ARSs and AIMPs were regarded as housekeeping molecules without additional functions. However, growing evidence indicates that ARSs and AIMPs are involved in tumorigenesis (Yum et al., 2016; Hyeon et al., 2019; Zhang et al., 2020a).

Di et al. found that isoleucyl-tRNA synthetase 2 (IARS2) acted as an oncogene in NSCLC by activating the protein kinase B (AKT)/mammalian target of rapamycin (mTOR) pathway (Di et al., 2019). IARS2 was highly expressed in NSCLC tissues, and silencing IARS2 could inhibit the activity of lung cancer cells and reduce the tumorigenicity of cancer cells in nude mice. Not only leucyl-tRNA synthetase (LRS) was significantly upregulated in lung cancer cell A549 but also its mRNA was highly expressed in primary lung cancer tissues (Shin et al., 2008). To explore the carcinogenic potential of the overexpression of LRS in lung cancer, Shin et al. knocked down the LRS in A549 cells and found that the growth and migration of cancer cells were significantly inhibited, indicating that this molecule played an important role in the development of lung cancer (Shin et al., 2008). Of note, AIMP2 had a tumor suppressor activity on lung cancer cells by Smad ubiquitination regulatory factors 2 (Smurf2) (Kim et al., 2016). AIMP2 was phosphorylated by transforming the growth factor-β (TGF-β)–activated p38MAPK, and the phosphorylated AIMP2 was separated from the MSC (Kim et al., 2016). Subsequently, the dissociated AIMP2 translocated to the nucleus and interacted with Smurf2 to exert its nuclear function. On the one hand, this interaction promoted the degradation of ubiquitin-mediated FUSE-binding protein (FBP) and thus downregulated c-Myc. On the other hand, it inhibited the binding of Smurf2 to chromosomal region maintenance 1 (CRM1), thereby reducing the nuclear export of Smurf2 to sustain TGF-β signaling (Kim et al., 2016). TGF-β signaling is involved in the regulation of a variety of cellular functions, including cell proliferation, differentiation, migration, and apoptosis (Siegel and Massagué, 2003), and its alteration can lead to human diseases, such as cancer (Massagué, 2008; Ikushima and Miyazono, 2010).

**FIGURE 2:**
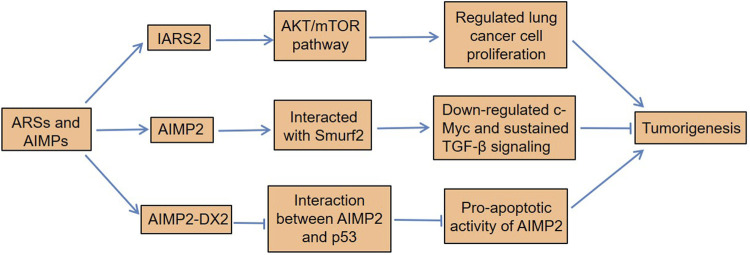
Dysregulation of ARSs and AIMPs in lung cancer. IARS2 regulates lung cancer cell proliferation by activating the AKT/mTOR pathway. AIMP2 interacts with Smurf2 to downregulate c-Myc and sustains TGF-β signaling, thereby inhibiting tumorigenesis. Moreover, AIMP2-DX2 reduces the proapoptotic activity of AIMP2 by competitive binding to p53, which induces lung tumorigenesis. ARSs, aminoacyl-tRNA synthetases; AIMPs, ARS-interacting multifunctional proteins; IARS2, isoleucyl-tRNA synthetase 2; Smurf2, Smad ubiquitination regulatory factors 2; TGF-β, transforming growth factor-β; AIMP2-DX2, AIMP2 lacking exon 2.

Meaningfully, the abundance of tyrosine-tRNA synthetase (TyrRS) and microtubule agglutinin cross-linking factor 1 (MACF-1) in lung adenocarcinoma tissue was higher than that in adjacent normal tissues (Zhou et al., 2013). Cox regression analysis found that patients with high TyrRS or MACF-1 expression had a significantly increased risk of death. Asparaginyl-tRNA synthetase (NARS), a class II ARS, had higher levels in lung adenocarcinoma than adjacent normal tissues, and it was positively correlated with lymph node metastasis (Hsu et al., 2016). Importantly, the downregulation of NARS could inhibit the growth and migration of adenocarcinoma cells (Hsu et al., 2016). Furthermore, methionyl-tRNA synthetase (MRS) had excessive mTORC1-related activities in NSCLC tissues, which played a vital role in tumor growth and spread (Kim et al., 2017a). Importantly, its overexpression was associated with poor clinical outcomes in patients with NSCLC. Interestingly, AIMP2-lacking exon 2 (AIMP2-DX2), a tumorigenic factor, is often upregulated in many cancers (Choi et al., 2011; Lim et al., 2020). AIMP2-DX2 is produced by alternative splicing, and it is highly expressed in human lung cancer cells and patient tissues. The ratio of AIMP2-DX2 to AIMP2 was positively correlated with the cancer stage, while it was negatively correlated with patient survival (Choi et al., 2011). This was because AIMP2-DX2 reduced the proapoptotic activity of AIMP2 by competing with p53 (Choi et al., 2011). The cells with higher levels of AIMP2-DX2 had a higher tendency to form anchorage-independent colonies, were more resistant to cell death, and increased the sensitivity of lung tumors (Choi et al., 2011). In all, certain ARSs not only contribute to the development of lung cancer but also serve as potential biomarkers for its diagnosis and prognosis (Figure 2).

As ARSs and AIMPs are closely related to the development of tumors, they may become potential targets for tumor treatment. LRS played an important role in activating the mTORC1 pathway and cell growth (Kim et al., 2019). Interestingly, the new LRS inhibitor BC-LI-0186 inhibited the mTORC1 signaling pathway by interacting with the RagD site of LRS, leading to the cytotoxicity of NSCLC cells and anticancer effects in the K-ras mouse lung cancer model (Kim et al., 2017b; Kim et al., 2019). These results provide a new therapeutic strategy for NSCLC. Moreover, Lim et al. found that the heat shock protein HSP70 (HSP70) was a critical factor which affected the level of AIMP2-DX2, and it was positively correlated with the level of AIMP2-DX2 in lung cancer cells (Lim et al., 2020). HSP70 could block the seven in absentia homolog 1 (Siah1)– mediated ubiquitination of AIMP2-DX2 by interacting with AIMP2-DX2, thereby maintaining the stability of AIMP2-DX2 and further enhancing AIMP2-DX2–induced cell transformation and cancer progression (Lim et al., 2020). Notably, BC-DXI-495 specifically inhibited the interaction of AIMP2-DX2 with HSP70, thereby inhibiting tumorigenesis (Lim et al., 2020). Another research discovered that AIMP2-DX2 also could prevent oncogene-induced apoptosis and senescence by directly binding to and inhibiting p14/ARF (Oh et al., 2016). However, the inhibition of DX2-p14/ARF interaction played an antitumor effect in lung cancer and delayed tumor progression (Oh et al., 2016). Taken together, although AIMP2-DX2 is a tumorigenic factor in the lung cancer progression, it can also be used as a treatment strategy for patients suffering from lung cancer.

## Conclusion and Future Perspective

Normally, tRNAs are considered to be housekeeping molecules with no additional functions. However, more and more evidence shows that tRNAs are related to various physiological and pathological processes (Lord et al., 2017; Liu et al., 2020; Zhu et al., 2020; Meng et al., 2021). As mentioned above, tRNA-associated metabolism plays a vital role in the development of lung cancer (Table 1). In recent years, many studies have analyzed the expression landscape of tRNAs, tRFs, tRNA modifying enzymes, and ARSs in cancer samples from The Cancer Genome Atlas (TCGA) (Pliatsika et al., 2018; Zhang et al., 2018; Hernandez-Alias and Benisty, 2020). For example, Telonis et al. found that i-tRFs were the richest tRFs among 32 human cancer types, and mt-tRNAs contributed to more tRFs than the nuclear ones (Telonis et al., 2019). There were associations between the identified tRFs and mRNAs, and the corresponding mRNAs usually belonged to the same biological processes, such as cell–matrix adhesion, receptor tyrosine kinase (RTK) signaling, and DNA and RNA metabolism. Importantly, many components of the MAPK signaling pathway were differentially related to tRFs between the sexes in lung adenocarcinoma, indicating that tRFs might play a role in underlying the sex disparities in the development of lung cancer (Telonis et al., 2019). Another TCGA-based analysis confirmed that the high expression of tRNA-ribosyltransferase 1 (QTRT1), an enzyme involved in the posttranscriptional modification of tRNAs, was associated with short overall survival in lung adenocarcinoma (Ma and He, 2020). Although these findings still need to be verified by *in vivo* and *in vitro* experiments, they greatly expand the research ideas of tRNA in the context of lung cancer.

**TABLE 1:** Roles of tRNA-associated metabolism in lung cancer.

tRNA-associated metabolism	Effects	Mechanisms	References
TERT	Promoted cancer cell proliferation	Regulated the expression of tRNA^Met^, tRNA^Arg^, and tRNA^Lys^, thus controlling protein synthesis in cancer cells	Khattar et al. (2016)
Mutations in mt-tRNAs, such as tRNA^His^, tRNA^Ala^, tRNA^Leu^, tRNA^Ser^, and tRNA^Thr^	Associated with lung tumorigenesis	—	Wang et al. (2015); He et al. (2016)
Dysregulation of tRNA levels	Used as variables to construct a prognostic model of lung adenocarcinoma and predict cancer-specific survival	—	Kuang et al. (2019)
ts-3676 and ts-4521	Involved in tumorigenesis	Not only acted as microRNAs but also served as piRNAs	Pekarsky et al. (2016)
ts-46 and ts-47	Affected lung cancer cell growth and survival	Inhibited the colony formation in lung cancer cells	Balatti et al. (2017)
tRF-Leu-CAG	Promoted cell proliferation and cell cycle in NSCLC	Increased the activity of AURKA	Shao et al. (2017)
FTSJ1	Suppressed the malignancy of NSCLC	By inhibiting the DRAM1 expression	He et al. (2020)
hTRM9L	Suppressed the proliferation, migration, and invasion of lung cancer cells	Inhibited the expressions of cyclin D1 and N-cadherin and promoted the expression of E-cadherin	Wang et al. (2018)
hDUS2	Contributed to tumorigenesis	Increased the translation efficiency by interacting with EPRS	Kato et al. (2005)
TRIT1	Cancer cells with TRIT1 gene amplification were more sensitive to arsenic trioxide	Involved in the expression of selenoproteins	Coll-SanMartin and Davalos (2021)
IARS2	Acted as an oncogene in NSCLC	By activating the AKT/mTOR pathway	Di et al. (2019)
LRS	Played an important role in the development of lung cancer	Promoted the growth and migration of cancer cells	Shin et al. (2008)
AIMP2	Had a tumor-suppressor activity on lung cancer cells	Interacted with Smurf2 to downregulate c-Myc and sustain TGF-β signaling	Kim et al. (2016)
TyrRS	High TyrRS expression increased the risk of death	—	Zhou et al. (2013)
NARS	Promoted the growth and migration of adenocarcinoma cells	—	Hsu et al. (2016)
MRS	Associated with tumor growth and spread	Had excessive mTORC1-related activities in NSCLC tissues	Kim et al. (2017a)
AIMP2-DX2	Induced lung tumorigenesis	Reduced the proapoptotic activity of AIMP2 through competitive binding to p53	Choi et al. (2011)
LRS	Associated with lung tumorigenesis	Activated the mTORC1 pathway	Kim et al. (2019)
AIMP2-DX2	Prevented oncogene-induced apoptosis and senescence	Through binding to and inhibiting p14/ARF	Oh et al. (2016)

Gu et al. found that tsRNAs, rRNA-derived small RNAs (rsRNAs), and yRNA-derived small RNAs (ysRNAs) in human peripheral blood mononuclear cells were differentially expressed between lung cancer, pulmonary TB, and control individuals (Gu et al., 2020). Of these, the levels of tsRNAs derived from tRNA^Ala^, tRNA^Asn^, tRNA^Leu^, tRNA^Lys^, and tRNA^Tyr^ were higher in lung cancer patients. Importantly, the researchers built a molecular signature based on 25 distinct ts/rs/ysRNAs, and this signature could precisely distinguish lung cancer patients from other subjects, indicating that tsRNAs might be used as potential biomarkers for lung cancer. Another research discovered that MRS and CD45 dual immunofluorescent staining had a good diagnostic performance for NSCLC patients with lymph node metastasis, and it could be used as a supplement to the routine cytology test (Lee and Kim, 2019). These findings indicate that certain tRNA fragments and ARSs may have great diagnostic values for lung cancer.

In addition, tRNA-related molecules are considered as therapeutic targets for lung cancer. Several studies have confirmed that tRNA derivatives can promote or inhibit the development of lung cancer (Balatti et al., 2017; Shao et al., 2017). Therefore, antisense molecules or mimetics targeting these tsRNAs can be used as potential small-molecule drugs. For example, tiRNA-Gly interacted with RNA-binding motif protein 17 (RBM17) to regulate alternative splicing, thereby promoting the proliferation and migration of papillary thyroid carcinoma (PTC) cells (Han et al., 2021). Notably, si-tiRNA-Gly significantly reduced the levels of tiRNA-Gly in PTC cells and inhibited their proliferation and migration. Another experiment found that the transfection of synthetic RNA mimetics of tRFs derived from tRNA^Glu^, tRNA^Tyr^, tRNA^Asp^, and tRNA^Gly^ could inhibit cancer metastasis to the lungs (Goodarzi et al., 2015). In addition, methyltransferase-like 1 (METTL1), a tRNA-modifying enzyme, was upregulated in lung adenocarcinoma tissues and inhibited autophagy in lung cancer cells through the AKT/mTORC1 pathway (Wang et al., 2021a). Conversely, HCC827 cells transfected with si-METTL1 showed enhanced autophagy, indicating that METL1 might be a promising target for the treatment of lung cancer. Interestingly, the antiparasitic drug pyrimethamine promoted the degradation of AIMP2-DX2 through ubiquitination, thereby suppressing the growth of H460 cells in xenograft mice (Kim et al., 2020). In conclusion, future research based on tRNA will help in understanding the pathogenesis of lung cancer and provide new ideas for its diagnosis and treatment.
